# The comparison of five low cost liquid formulations to preserve two phosphate solubilizing bacteria from the genera *Pseudomonas* and *Pantoea*

**Published:** 2016-12

**Authors:** Sanaz Goljanian-Tabrizi, Soleiman Amiri, Donya Nikaein, Zeinab Motesharrei

**Affiliations:** Applied Microbiology Research Group, Academic Center for Education Culture and Research (ACECR), Tehran Branch, Tehran, Iran

**Keywords:** Phosphate solubilization, *Pseudomonas putida*, *Pantoea agglomerans*

## Abstract

**Background and Objectives:**

Phosphorus is one of the low bioavailable macroelements. Use of microorganisms in biofertilizers could release phosphorus from insoluble compounds. *Pseudomonas putida* P13 and *Pantoea agglomerans* P5 are well recognized for application as phosphate solubilizing bioinoculants and are used as solid carrier based. Liquid bioinoculants are preferred for economizing production process and longer shelf-life.

**Materials and Methods:**

Five low cost liquid formulations were examined. Formulations 1, 2 and 3, were phosphate buffer, 0.2% and 0.5% KNO_3_ dissolved in phosphate buffer, respectively. Formulation 4 was nutrient broth containing 4% glycerol and formulation 5 was diluted nutrient broth containing 4% glycerol. Survival (cfu) and phosphate solubilization index (*SI*) were evaluated after 3 months.

**Results:**

Considering strain P5, increase in KNO_3_ concentration decreased preserving ability. While using KNO_3_ at 0.2% was accompanied with reaching maximum *SI* level. Overall, less nutritious formulations (1 and 5) provided maximum preserving ability without bioactivity loss. In the case of strain P13, maximum survival obtained in formulations 2 and 3, whereas *SI* level decreased. Preserving ability in formulations 1, 4 and 5 was similar but less nutritious formulations (1 and 5), improved bioactivity.

**Conclusion:**

The results introduced two formulations of 1 and 5 as economically efficient liquid bioinoculants for *Pseudomonas putida* and *Pantoea agglomerans.*

## INTRODUCTION

Nitrogen, phosphorus and potassium, are among the macronutrients which are required in large quantities for plants (1). Due to poor availability of phosphorus in many agricultural soils (2) biofertilizers could be used to release phosphorus from inorganic and organic phosphorus compounds (3). A biofertilizer (microbial culture or bioinoculant) is composed of live or latent microorganisms capable to augment the bioavailability of nutrients to the host plant (4).

*Pseudomonas putida* strain P13 and *Pantoea agglomerans* strain P5 are well known as effective phosphate solubilizing bacteria of related bioinoculants in Iran (5). Phosphatase enzyme produced by strain P13 and weak acids excreted by strain P5, in combination, hydrolyze mineral and organic phosphate compounds, turning phosphorus into bioavailable and absorbable forms for plants. These two strains are adapted to various climates including extreme climatic conditions of Iran (5, 6). Currently, the incorporation of the strains P13 and P5 into biofertilizers is in the form of carrier-based inoculant (6). Promotion of agricultural modernization necessitates production of liquid bioinoculants. Factors associated with potential benefits of liquid bioinoculants include: handling comfort, bioefficacy, low contamination and long-term storage (7). Composition, sustainability at ambient temperature, and maintenance of bioactivity in the desired duration, are significant criteria which determines the quality and cost effectiveness of a liquid biofertilizer.

The current research was carried out to compare different liquid formulations proposed in literatures for maintenance of *Pseudomonas strains* and *Pantoea agglomerans* under room temperature and to verify the possibility of introducing an effective and economic liquid biofertilizer to be used in agriculture, replacing the solid formulation.

## MATERIALS AND METHODS

### Bacterial strains and revival

Strains P5 and P13 were obtained from microbial culture collection of Iranian Academic Center for Education, Culture, and Research (ACECR), Tehran branch, Department of Applied Microbiology. Recovery of the two phosphate solubilizing bacteria (PSB) was made by sub-culturing few drops of stored bacterial stocks at −70°C onto nutrient agar medium. Plates were incubated at 30°C until growth was observed.

### Liquid formulations

For prolonged storage studies of strains P13 and P5 at ambient conditions, five different formulations were selected according to former studies (7–10) with minor modifications (Table 1). Isotonic phosphate buffer containing: KH_2_PO_4_ 15.44 μM, NaCl 1.55 mM, Na_2_HPO_4_ 27.09 μM (8) was used to compare the quality of long-term buffer maintenance in the presence (0.2% and 0.5%) or absence of KNO_3_. Tap water was used for preparation of the phosphate buffers. The formulations were prepared in 50 ml polypropylene falcon tubes at 1/2 volume and sterilized at 121°C for 15 minutes.

**Table 1.** Compositions of used formulations and related modifications.

**Formulation no.**	**Formulation composition**	**Modification**	**References**
**1**	Isotonic phosphate buffer	Optionally bufferic ions of NH_4_H_2_PO_4_ replaced by KH_2_PO_4_	Liao and shollenberger, 2003
**2**	0.2% KNO_3_ dissolved in isotonic phosphate buffer	Optionally bufferic ions of NH_4_H_2_PO_4_ replaced by KH_2_PO_4_	Vandenberg et al, 1997, Vandenberg, 2003
**3**	0.5% KNO_3_ dissolved in isotonic phosphate buffer	Optionally bufferic ions of NH_4_H_2_PO_4_ replaced by KH_2_PO_4_	Vandenberg et al, 1997, Vandenberg, 2003
**4**	Nutrient broth containing 4% glycerol	2% increase in glycerol concentration	Manikandan et al, 2010
**5**	Diluted nutrient broth containing 4% glycerol	Nutrient broth diluted with 3 volume distilled water and 2% increase in glycerol concentration	Manikandan et al, 2010

### Liquid bioinoculant preparation

For preparation of liquid bioinoculants, a loopful of 24 hours culture of the strains P13 and P5 in nutrient agar medium was transferred into nutrient broth and incubated at 30°C, 150 rpm for 14 hours (when the cells were still in logarithmic phase of growth). Cultures were then centrifuged at 5000 rpm for 15 minutes at 30°C and cell mass was collected and washed twice with equal volume of normal saline solution, subsequently. The cell mass was added to liquid formulations under sterile conditions (11).

The number of cells in each formulation was adjusted to 2 × 10^8^ cfu/ml for strain P5 and 1 × 10^7^ cfu/ml for strain P13 by measuring the 0.5 optical density at 540 nm wavelength OD_(540nm)_. These values are within the range of bacterial cell number allowed for a biofertilizer according to the plant protection organization of Iran. The samples were perfectly sealed and stored at 25°C in dark conditions.

### Survival of PSB strains in different liquid formulations

The assessment of viable populations of the two strains was made at monthly interval for three months. Serial four-fold dilutions in normal saline solution were made from each sample. An aliquot of 100 μl from each dilution was dropped on nutrient agar medium as the spread plate technique. The number of colony forming units (cfu) was determined after 24 hours of incubation (12).

### Phosphate solubilization index of liquid formulations

Phosphate solubilizing ability of the formulated strains was estimated after three months of storage. For this purpose, preserved bacterial solutions were centrifuged at 5000 rpm for 15 minutes at 30°C and biomass was collected. In order to do a precise comparison between maintained samples, the OD value of 0.5 at 540 nm wavelength, was chosen. Therefore, the recovered biomass was dissolved in ringer solution until an OD_(540nm)_ value of 0.5 was attained. 25 μl of each bioinoculant (at the OD_(540nm)_ value of 0.5) was spotted onto plates of Sperber′s agar medium to evaluate hydrolysable phosphorus from tri-calcium phosphate qualitatively by forming the clear zone in the opaque medium (13). Colony diameter and clear zone diameter were measured after incubating for seven days at 30°C. Phosphate solubilization index (*SI*) was calculated as follows (14):

$$\mathrm{SI}=\frac{\text{colony}\;\text{diameter}\;+\;\text{halo}\;\text{zone}\;\text{diameter}}{\text{colony}\;\text{diameter}}$$

Phosphate solubilization indexes of 24 hours cultures of strains P13 and P5 at OD_(540 nm)_ value of 0.5 were calculated and used as control. The same incubation conditions were applied for preserved bacteria and control.

### Statistical analysis

The assays were calculated as an average of three independent experiments. Data were analyzed using SPSS version 21. One-way analysis of variance (ANOVA) and Tukey test were used to examine values. P value <0.05 was considered significant.

## RESULTS

### Ability of formulations to preserve bacterial population

The ability of each formulation to preserve each single bacterial strain was evaluated over a three month period. Data related to population dynamics of strains P5 and P13, are presented in Table 2.

**Table 2.** Effect of storage at room temperature on population size of strains P5 (initially containing 2 × 10^8^ cfu/ml) and P13 (initially containing 1 × 10^7^ cfu/ml) in liquid formulations. Statistical significances of data at the third month are presented.

**Formulation no.**	**1^st^ month**	**2^nd^ month**	**3^rd^ month**	**1^st^ month**	**2^nd^ month**	**3^rd^ month**

	**Strain P5**			**Strain P13**		
**1**	6 × 10^6^	3 × 10^6^	5 × 10^6 a^	2 × 10^6^	2.4 × 10^6^	3.75 × 10^6 a^
**2**	5.6 × 10^6^	3.5 × 10^6^	3.25 × 10^6 bc^	4.8 × 10^7^	4.2 × 10^7^	2 × 10^7 b^
**3**	1 × 10^6^	2.9 × 10^6^	2.5 × 10^6 c^	2.1 × 10^7^	3.5 × 10^7^	2 × 10^7 b^
**4**	4.3 × 10^7^	2 × 10^7^	4 × 10^6 ab^	2.7 × 10^6^	4.6 × 10^6^	4 × 10^6 a^
**5**	3 × 10^7^	1 × 10^7^	5 × 10^6 a^	4.7 × 10^6^	3 × 10^6^	2.5 × 10^6 a^

* Unidentical letters represent significant differences between 3 months in each formulation

Strain P5 with the initial population of 2×10^8^ cfu/ml, underwent a reduction in number in all formulations after one month of storage. However, there was no significant reduction in the number of bacteria afterwards. Formulations 2 and 3 induced a reduction in cell number higher than other formulations at the end of the storage period. The simple phosphate buffer tended to preserve better the number of cells in comparison to phosphate buffers containing potassium nitrate (5 × 10^6^ cfu/ml). Furthermore, increasing the concentration of potassium nitrate in the formulation provoked a further reduction of bacterial population (3.25 × 10^6^ cfu/ml and 2.5 × 10^6^ cfu/ml for formulations 2 and 3, respectively).

Estimating the influence of nutritious formulations (formulations 4 and 5) on P5 viability, better maintaining ability by formulation of undiluted nutrient broth (formulation 4) was not longer than two months and overall better performance was provided by less nutritious formulation (formulation 5) after three months of storage.

The results of different formulations for preserving strain P5 indicated that both of the formulations 1 and 5 supported the highest maintaining ability and protected population maintenance of 5 × 10^6^ cfu/ml.

The highest survival rate at the end of the storage of strain P13 was assured by formulations 2 and 3 (2 × 10^7^ cfu/ml), but with the limitation of cell proliferation for duration of one month (in formulation 2) and two months (in formulation 3) after storage. Statistically no significant difference was observed among formulations 1, 4 and 5 (Table 2).

### Ability of formulations to preserve phosphate solublilizing activity

Phosphate solublilizing activity of strains P5 and P13 was assessed after three months in comparison to enzymatic activity of freshly cultured bacteria (Table 3).

**Table 3.** *SI* of the strains P5 and P13 after three months of storage with different formulations in comparison to freshly cultured bacteria.

**Formulatio no.**	***SI* level**	

	**strain P5**	**strain P13**
**1**	3.81^ac^	3.86^a^
**2**	3.98^b^	2.43^b^
**3**	3.86^a^	2.47^b^
**4**	3.74^c^	2.41^b^
**5**	3.81^ac^	3.81^a^
**Control**	3.81^ac^	3.5^c^

Bacteria of strain P5 with formulations 1 and 5 showed a *SI* level equal to that of fresh culture after three months (*SI*_ control_ = 3.81). Compared to the control, the addition of potassium nitrate to the phosphate buffer of formulations 2 (*SI* = 3.98) and formulation 3 (*SI* = 3.86) enhanced *SI* ability. Formulation 4, with undiluted nutrient broth, resulted into the lowest index of P solubilization (*SI* = 3.74).

In the case of strain P13 as compared to the control (*SI*_ control_ = 3.5), formulations 1 and 5 enhanced the bacterial ability to solubilize phosphate. The highest *SI* for P13 was obtained with the simple phosphate buffer of formulations 1 (*SI* = 3.86) and diluted broth of formulation 5 (*SI* = 3.81). The presence of potassium nitrate in the phosphate buffer formulation reduced *SI* (contrary to the results observed for strain P5 in which maximum *SI* was obtained in the presence of 0.2% KNO3). Formulations 2, 3, and 4, induced a decline on phosphatase activity.

## DISCUSSION

Considering both bacteria as Gram negative and facultative anaerobes, efficiency of each individual formulation was examined for each strain, separately. Maximum population of strain P5 and one of highest *SI* of strain P13, were obtained with formulation 1 based on a simple phosphate buffer. Additionally, in this formulation, phosphate solubilization index of strain P5 was equal to *SI* of control and population size of strain P13 was also high. Population sustainability in formulation 1 is in accordance with the data of Liao and Shollenberger (2003). In their study, it was evidenced that the majority of pathogenic bacteria were able to survive in either water or phosphate buffer after 30 weeks of preservation at room temperature, allowing to hypothesize that human and plant pathogenic bacteria can survive in water or phosphate buffer for several years.

Maximum preserving ability during three months of storage for strain P13 was assured by phosphate buffers containing potassium nitrate. The formulations were confined by reducing *SI* level and increasing cell number for at least about one month after storage of the strain P13. Formulation 2, contributed to maximum *SI* of strain P5 and formulation 3 decreased population level of the strain to its minimum. Our findings about the ability to sustain the *Pseudomonas* population size of the phosphate buffer based formulations are in line with findings of Vandenbergh and coworkers (9, 10) who found that after about one month, stored *Pseudomonas* in buffer containing potassium nitrate showed 58% more viability than bacteria stored in buffer lacking potassium nitrate. They suggested that low levels of alkali metal nitrate (0.1–0.5%) were sufficient to maintain *Pseudomonas* for up to 160 days or more at room temperature.

Numerous studies have described glycerol amendment for improving shelf life of *Pseudomonas* (and other microorganisms meanwhile) at room temperature (15–19). Glycerol has the advantages of being a colorless and odorless liquid. It was added to the formulations 4 and 5 as osmoprotectant to extend bacterial viability, since it holds large amounts of water and enhance protection of cells under desiccation (7, 20). Therefore risk of significant reduction in water activity in nutrient broth medium was not confronted. Noticing that a liquid carrier should be low in viscosity in order to be pumped easily, we used glycerol at only 4% weight/volume ratio (21).

The maintained population size for both strains was acceptable in formulation 4, but *SI* dropped to low efficacy in both strains. Formulation 5 enhanced solubilization index in comparison to the control for strain P13 and maintained it equal to the control for strain P5, for which also maximum population was yielded (maximum population for strain P5 was also yielded in formulation 1).

The obtained results in formulations 4 and 5 can be compared with the findings of Manikandan et al. (2010). Nutrient broth medium containing 2% glycerol amendment has been defined as an appropriate formulation for *P. fluorescens* preservation at room temperature. The reported potent bacterium maintained its antagonistic activity until 150 days without any remarkable reduction in bioactivity (7).

The formulation 5 fitted with two main positive features; keeping both strains bioactive along with promotion of strain P5 to highest population density. However, the formulation was not so effective in maintaining the P13 strain population. Considering the direct relationship of plant growth promotion efficacy with the number of inoculated microorganisms, the population decline of strain P13 in formulation 5 could be counteracted with inoculation of more bacterial biomass.

Adhesives (any locally available nontoxic sticky material) can be added to water or microbial culture broth of inoculant formulation at on seed level. Sucrose is a common additive which can be used as an extender or an adhesive in liquid formulations of *Pseudomonas*. It is better to add extenders at on seed level or at sowing time (22–25). *Pantoea agglomerans* strain J49, has been essayed as a promising phosphate solubilizing bacterium. Utilization of the formulation as an effective bioinoculant, was ameliorated by preparation of a 20% sucrose solution (as adherent) for on seed level use (26). Addition of 0.1% methyl cellulose (as an adhesive and protectant) to bioactive *P. aeruginosa* liquid medium for on seed application of the bioinoculant, is reported (27).

In prior studies, it has been demonstrated that mixed inoculation of the two phosphate solubilizing bacterial strains P5 and P13, can be performed (6).

In conclusion it would be possible to utilize formulations 1 and 5 for production of efficient liquid bioinoculants composed of single strain and both strains tested.
